# Lymphatic delivery of etanercept via nanotopography improves response to collagen-induced arthritis

**DOI:** 10.1186/s13075-017-1323-z

**Published:** 2017-05-31

**Authors:** Melissa B. Aldrich, Fred C. Velasquez, Sunkuk Kwon, Ali Azhdarinia, Kenneth Pinkston, Barrett R. Harvey, Wenyaw Chan, John C. Rasmussen, Russell F. Ross, Caroline E. Fife, E. M. Sevick-Muraca

**Affiliations:** 1 The Center for Molecular Imaging, The Brown Foundation Institute of Molecular Medicine, The University of Texas Health Science Center, Houston, TX 77030 USA; 2 Department of Biostatistics, The School of Public Health, The University of Texas Health Science Center, Houston, TX 77030 USA; 3 Kimberly Clark Corporation, Atlanta, GA USA; 4 The Wound Care Clinic, CHI St. Luke’s Health, The Woodlands Hospital, The Woodlands, TX 77382 USA

**Keywords:** Lymphatic pumping function, Nanotopography, Delivery systems, Route of administration, Etanercept, Near-infrared fluorescence lymphatic imaging

## Abstract

**Background:**

Evidence suggests lymphatic function mediates local rheumatoid arthritis (RA) flares. Yet biologics that target the immune system are dosed systemically via the subcutaneous (SC) administration route, thereby inefficiently reaching local lymphatic compartments. Nanotopography has previously been shown to disrupt tight cellular junctions, potentially enhancing local lymphatic delivery and potentially improving overall therapeutic efficacy.

**Method:**

We first characterized nanotopography (SOFUSA™) delivery of an anti-TNF drug, etanercept, by comparing pharmacokinetic profiles to those obtained by conventional SC, intravenous (IV), and intradermal (ID) routes of administration, and assessed uptake of radiolabeled etanercept in draining lymph nodes (LNs) in single dosing studies. We then compared etanercept efficacy in a progressive rat model of collagen-induced arthritis (CIA), administered systemically via SC route of administration; via the regional lymphatics through ID delivery; or through a nanotopography (SOFUSA™) device at 10, 12, and 14 days post CIA induction. Measurements of hind limb swelling and near-infrared fluorescence (NIRF) imaging of afferent lymph pumping function and reflux were conducted on days 11, 13, and 18 post CIA induction and compared to untreated CIA animals. Univariate and multivariate analysis of variance were used to compare the group differences for percentage swelling and lymphatic contractile activity.

**Results:**

Even though all three modes of administration delivered an equal amount of etanercept, SOFUSA™ delivery resulted in increased lymphatic pumping and significantly reduced swelling as compared to untreated, ID, and SC groups. Pharmacokinetic profiles in serum and LN uptake studies showed that using the nanotopography device resulted in the greatest uptake and retention in draining LNs.

**Conclusions:**

Locoregional lymphatic delivery of biologics that target the immune system may have more favorable pharmacodynamics than SC or IV administration. Nanotopography may provide a more efficient method for delivery of anti-TNF drugs to reverse impairment of lymphatic function and reduce swelling associated with RA flares.

**Electronic supplementary material:**

The online version of this article (doi:10.1186/s13075-017-1323-z) contains supplementary material, which is available to authorized users.

## Background

Rheumatoid arthritis (RA) is a chronic inflammatory joint disease and one of several immune-mediated inflammatory disorders with etiology that is not well-understood. RA is characterized by inflammation in the synovial membrane, cartilage, and bone, where accumulation of immune cells is observed. Patients experience severe pain, swelling, and erosion of the joints that leads to joint deformity and disability. In patients who are unresponsive to non-steroidal anti-inflammatory drugs or disease modifying anti-rheumatic drugs, a number of biologic agents (antibodies or recombinant proteins) that block activity of inflammatory mediators or signaling molecules provide another line of defense [1]. Examples include biologics that: (1) deplete B-cells that produce autoantibodies characteristic of RA or (2) block co-stimulatory signals needed to activate T-cells within the lymph nodes (LNs). In the latter case, biologic targets are pro-inflammatory cytokines such as anti-tumor necrosis factor (TNF), interleukin-6 (IL-6), and interleukin-1 (IL-1) that are secreted by macrophages and play important roles in T-cell activation. Interestingly, while the sites of action of these cytokine and signaling molecules may be in regional LNs and lymphatic vasculature, biologics that target the immune system are provided through intravenous (IV) or subcutaneous (SC) routes that may not efficiently reach the lymphatics.

The lymphatic vasculature is a unidirectional highway for the transit of immune cells, activation of T cells and B cells, and return of activated immune cells, cellular waste, and excess fluid to the blood vasculature [2]. The lymphatic vasculature starts with initial lymphatic capillaries beneath the epidermis that surround most organs and the synovium. These capillaries are composed of single layers of lymphatic endothelial cells with no basement membrane. Immune cells, waste products, and fluid enter between specialized button-like, lymphatic endothelial cell junctions [3] and are propelled through mature conducting lymphatic vessel segments called lymphangions, which are bounded by valves that open and close in concert with lymphatic smooth muscle contraction to efficiently pump lymph proximally and through regional LNs, before emptying into the blood stream. Entrance of exogenous agents into the lymphatic vasculature can occur via an intradermal (ID) injection into space occupied by the initial lymphatics under the epidermis, or to a lesser extent, into the high endothelial venules of LNs following IV injection. SC administration may enable indirect entry into both venous and lymphatic vasculatures, but is limited from efficient uptake from the interstitium into blood vessels due to the intact tight endothelial junctions and glycocalyx that severely regulates the flow of high molecular-weight molecules across the blood vessel wall [4]. SC administration thus misses the ID space beneath the epidermis containing the initial lymphatics that efficiently take up macromolecules. As a consequence, SC administration is associated with reduced bioavailability and impaired systemic and lymphatic delivery.

As reviewed elsewhere [5, 6], several recent clinical and experimental observations implicate the lymphatic vasculature in the pathogenesis of RA. Lymphedema and RA are known comorbidities [7], and lymphangiography studies show that RA patients have abnormal lymphatic vasculature with extensive dermal reflux, consistent with lymphedema [8]. In addition, enlarged and greater numbers of popliteal LNs, and increased synovial fluid volumes in the knees of RA patients, have been reported along with elevated pro-inflammatory cytokine levels in lymph-draining synovial joints, as compared to levels found in serum [9]. In animal studies, elevated levels of the pro-inflammatory and pro-lymphangiogenic cytokine vascular endothelial growth factor C (VEGF-C) have been found in the joints of tumor necrosis factor transgenic (TNF-Tg) animals, which mimic human RA pathogenesis [10]. TNF-Tg animals experience B-cell expansion in draining LNs with concomitant collapse and loss of afferent lymph vessel pumping, as measured by non-invasive near-infrared fluorescence (NIRF) lymphatic imaging [11], and proinflammatory cytokines TNF-α and IL-1β cause lymphatic vessel dilation and arrest of the lymphatic pump that can be rescued by ID-administered inducible nitric oxide (iNOS) inhibitors [12]. Because the draining lymphatic vasculature and LNs have been shown to mediate adaptive immune responses [13] and may mediate RA flares, [14] delivery of therapeutic agents through the lymphatic compartment could be expected to mediate a systemic response with an enhanced effect on local disease manifestation.

While systemic anti-TNF therapy is considered successful in the treatment of RA, a substantial percentage of patients (~30 − 40%) fail to respond, exposing them to unnecessary adverse events while disease progresses [15, 16]. Similar failure rates are reported for other biologics administered for RA, and efforts to identify biomarkers that could predict response rates have failed [17].

Given the importance of the draining lymphatics in RA, we sought to determine whether nanotopography-directed lymphatic delivery of an anti-TNF drug, etanercept, could result in improvement of lymphatic pumping function and reduced swelling in the collagen-induced arthritis (CIA) model of RA progression. We first characterized the pharmacokinetic (PK) profiles and measured the accumulation of etanercept in draining LNs using nanotopography, ID, and SC local administration, and using systemic IV administration. Because CIA animals have varying disease severity that could impact measurements, we performed these measurements in normal animals. Then in CIA animals, we monitored hind limb swelling and lymphatic pumping function using NIRF lymphatic imaging [18] before, during, and following a course of etanercept administered SC, ID, or with a nanotopographic device that infused drug directly into the intradermal space containing the initial lymphatics. We show that lymphatic delivery of etanercept predominated with nanotopography (SOFUSA™) and resulted in improved pumping function of afferent lymphatic vessels and reduced hind limb swelling in CIA animals when compared to animals treated with SC-administered or ID-administered etanercept.

## Methods

### General procedures and reagents

#### Animals and housing

Male Lewis rats, each weighing approximately 350 g, were obtained from Charles River (Wilmington, MA, USA) and housed in an Association for Assessment and Accreditation of Laboratory Animal Care-approved facility, according to institutional guidelines. All animal protocols were reviewed and approved by the Institutional Animal Care and Use Committee at the University of Texas Health Science Center-Houston. Animals used for CIA induction, PK, and LN uptake studies were ear-tagged prior to handling.

#### Reagents

All reagents were analytical grade and used without further purification unless otherwise stated. Etanercept (Enbrel, Amgen, CA, USA) was received in 50 mg/ml dosage form and diluted to 10 mg/ml for dosing.

#### Radiolabeled etanercept

Radiolabeled ^64^Cu-NODAGA-etanercept was used to validate the amount of drug delivered by SOFUSA™ and the amount of etanercept in LNs following the different routes of administration. To synthesize NODAGA-etanercept, a 10-mg aliquot of etanercept was buffer-exchanged into 0.1 M sodium phosphate buffer (pH 8.3) and reacted with a 20-fold molar excess of NODAGA-NHS (Chematech, Dijon, France) for 4 h at room temperature, then kept in a fridge at 4 °C overnight. The reaction was purified with Zeba desalting spin columns (Thermo Scientific, Rockford, IL, USA) and collected in PBS for radiolabeling and immunoreactivity studies. The average number of NODAGA molecules per protein was quantified by isotopic dilution as previously described [19] and was 1.4. Solution concentration was determined by ND-1000 spectrophotometer (NanoDrop, Wilmington, DE, USA). Retention of TNF-α binding affinity of NODAGA-etanercept bioconjugate was validated by comparing ELISA binding of naïve etanercept with NODAGA-etanercept. For final radiolabeling prior to administration, 1 mg of NODAGA-Enbrel was then buffer-exchanged into 0.1 M NaOAc (pH 6) and reacted with 50 μl of ^64^Cu in buffer. ^64^CuCl_2_ was produced by Washington University School of Medicine (St. Louis, MO, USA). After incubation at room temperature for 1 h, 3 μL of 10 mmol EDTA was added, and samples were incubated for an additional 15 minutes. The samples were Zeba-purified and collected with 80.7 ± 8.3% radiochemical yield and >99% radiochemical purity, as determined by high-performance liquid chromatography (HPLC) analysis. For radiolabeled etanercept dosing, we added a 9:1 stoichiometric ratio of naïve, unlabeled etanercept to radiolabeled etanercept, yielding a 10-mg/mL solution for dosing. The radioactivity of the dosing solution was measured using CRC-25R dose calibrator (Capintec, Inc., Florham Park, NJ, USA).

#### Nanotopographic device for drug delivery (SOFUSA™)

**Fig. 1:**
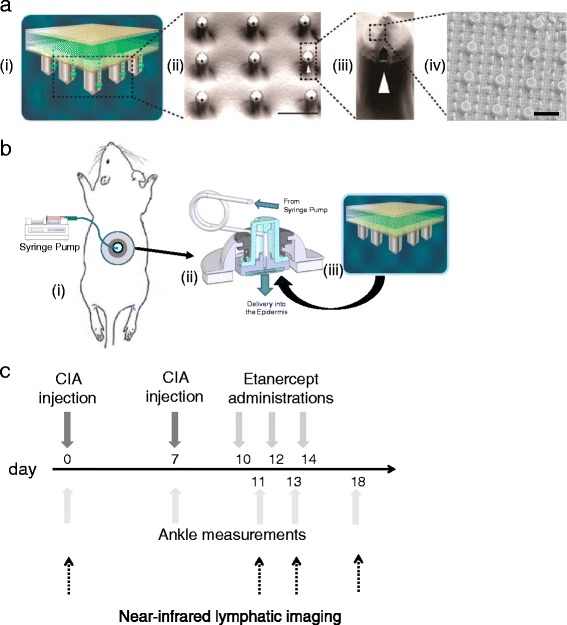
**a** The SOFUSA™ nanotopographical device. (*i*) Microfluidic fluid block with a perforated attachment adhesive (*tan*), microfluidic distributor (*green*), perforated attachment adhesive (*yellow*), and silicon microneedle array (*gray*). Each microneedle is 350 μm long and 110 μm wide, with a 30-μm hole located off-center, through which the drug flows out of a total of 100 microneedles (*ii*) Scanning electron microscopy (SEM) image of nanotopographic film heat formed over the silicon microneedles (*scale bar* represents 300 μm); (*iii*) SEM of individual microneedle, and (*iv*) SEM image of the nanostructures on each microneedle (*scale bar* represents 3 μm). **b** Administration procedure for the SOFUSA™ nanotopographical device. (*i*) Location of SOFUSA™ on the rat dorsal region during drug delivery experiments; (*ii*) cross-sectional illustration of the complete SOFUSA™ device, and (*iii*) microfluidic fluid block as defined in panel **a**. **c** Timeline for induction, measurements, and treatments of animals with collagen-induced arthritis

SOFUSA™ is a microneedle drug delivery device with a nanotopographical imprinted polyether, ether, ketone film heat-formed over each microneedle on the array (Fig. 1a). The nanotopographical film-microneedle combination has been found to increase permeability through the skin epidermis layer by remodeling tight junction proteins initiated via integrin binding to the nanotopography [20]. The increased permeability enables SOFUSA™ to deliver therapeutic drug levels and control targeting to the lymphatic system based on the delivery occurring between the stratum corneum and initial lymphatic capillaries, and the ability to change the number of microneedles and the microneedle flowrate.

Twenty-four hours prior to SOFUSA™ administration, rats were anesthetized with isofluorane and the dorsal region was shaved and covered with depilatory cream (Nair Sensitive) for 8 minutes. The cream was then wiped off with warm, wet paper towels, followed by alcohol wipes. SOFUSA™ was then applied to the dorsal region using a plastic shell with a skin adhesive (Fig. 1b). A hand-held applicator was then placed over the plastic shell to insert the microneedles into the skin. The operation of the device was as follows. The applicator strikes the microneedles with a post traveling at a velocity of 6 m/s. There are a total of 100 microneedles over the applicator area of 66 mm^2^. With the microneedles inserted in the skin, the syringe pump is started to deliver the agent. In these studies, the syringe pump was set at a constant rate of 100 μL/h and was run for 1 h to deliver the 1 mg etanercept. The etanercept dosing was 10 mg/mL.

### Characterization of etanercept delivery via SOFUSA™, IV, SC, and ID routes of administration

#### PK profiles

In order to characterize differences in etanercept delivery by the different routes of administration, we used normal rats to determine the PK profiles following a single dose of 1 mg etanercept in 100 uL delivered via SOFUSA™ (*n* = 6), conventional IV (in the tail vein, *n* = 6), SC (dorsolateral injection at the same site as the SOFUSA™ application, *n* = 4), or ID injections (symmetrical dorsolateral injections 2 × 50 uL for a total dose of 1 mg etanercept, *n* = 6). Other than the SOFUSA™ delivery, all administration was conducted using a 31-gauge needle. At 2, 4, 8, 12, 24, and 36 h after administration, animals were anesthetized under isoflurane, and 200 μL of blood was drawn from the jugular vein. The etanercept concentration in serum was quantified using the Etanercept ELISA Kit (ABIN: 1540251) (Matriks Biotechnology Co., LTD., Ankara, Turkey). Optical density was measured at 450 nm using Thermo Scientific Multiskan EX (Thermo Fisher Scientific, Waltham, MA, USA).

### Measurement of LN uptake of etanercept via different routes of administration in normal rats

In order to determine the etanercept delivered to the LNs, we administrated the radiolabeled etanercept solution via SOFUSA™ (1 mg etanercept in 100 uL), IV (1 mg etanercept in the tail vein in 100 uL), SC (1 mg etanercept in the dorsolateral side at the same site as the SOFUSA™ application, 100 uL), and ID (1 mg total etanercept in two 50-μL injections). Animals were harvested at 12 and 36 h after administration, and the left/right axillary and inguinal LNs were collected, weighed, and counted for radioactivity using a 2480 Wizard^2^ automatic gamma counter (PerkinElmer, Waltham, MA, USA). The time-corrected radioactivity was then used to compute the μg/mL of tissue etanercept concentration from the specific radioactivity of the dosing solution with animals that were euthanized at 12 h (*n* = 4, SOFUSA™; *n* = 6, IV; *n* = 6, ID; and *n* = 6, SC) and at 36 h (*n* = 6, SOFUSA™; *n* = 6, IV; *n* = 6, ID; and *n* = 6, SC).

#### Quantification of etanercept delivered

In order to validate the amount of etanercept delivered, we measured the radioactivity in the SOFUSA™ device and tubing before and after the 1-h infusion. Measurement was conducted using the dose calibrator. We computed the amount of etanercept as the difference in the time-corrected radioactivity before and after administration,. We similarly computed the amount of etanercept delivered via conventional syringes using the same method, with the expectation that the amount delivered matched that determined from radioactivity balance.

To directly visualize SOFUSA^TM^ delivery, 100 μL of 645 μM indocyanine green (ICG) (Akorn, Inc.) in sterile saline was delivered over 1 h using a syringe pump (model NE-300, SyringePump.com) connected to the SOFUSA™, which was applied to the dorsal surface on the right side of the rat. NIRF imaging was conducted as described subsequently.

### Treatment of CIA animals with etanercept administered by SOFUSA™, SC, and ID routes of administration

#### CIA induction, hind limb measurement, and measurements

Type II porcine collagen (Chondrex, Inc. catalog #20031), solubilized in 0.05 N of acetic acid in sterile water at a concentration of 2 mg/mL was emulsified with an equal volume of incomplete Freud’s adjuvant (Chondrex, Inc. catalog #7002) using homogenization at 35,000 rpm (Omni International homogenizer TH, homogenizer probe #32750): 100 μL emulsion was injected subcutaneously at the base of the tails on both sides for initial administration (day 0, 200 μL of emulsion total), and then again, ***7*** days later, on the right side only, for booster administration (day 7, 100 μL of emulsion total). Hind limb swelling usually became evident at day 14. Hind limb swelling was assessed by caliper measurements of the rear ankle cross (side-to-side) and oblique (front-to-back) dimensions. The two measurements for each hind limb were multiplied together for assessment of swelling as done in other studies [21] and percent change from baseline was computed. These measurements and lymphatic imaging (as described subsequently) were performed on days 0, 7, 11, 13, and 18, in *the* early stages of CIA before the onset of joint destruction [22]. Four groups of animals were studied: (1) untreated (*n* = 20), (2) treated with etanercept given by SC administration (*n* = 20, 1 mg in 100 μL), (3) treated with etanercept given by ID administration (*n* = 20, two administrations of 0.5 mg in 50 μL), and (4) treated with etanercept given via SOFUSA™ (*n* = 18, one administration of 1 mg in 100 μL delivered). Figure 1c shows the timeline for induction, measurement, and treatment of CIA animals. *W*e did not include IV treatment of CIA animals *b*ecause etanercept is not administered IV in RA patients, and because repetitive IV administration in animals is invasive.

#### Imaging of lymphatic pumping function

Rats were anesthetized with isoflurane and shaved before imaging: 10 μL of 625 μM ICG was then injected ID with a 31-gauge needle/syringe (BD #328438, Fisher Scientific) at the base of the tail and on the dorsal side of the paw on both the right and left sides of the rats to perform NIRF imaging of the lymphatics. NIRF images were collected with a custom-built system that employed illumination of tissue surfaces with 785-nm light from a laser diode (85 mA and 80 mW, DL7140-201, Sanyo) that was diffused to cover a circular area approximately 8 cm in diameter [23]. Fluorescent light generated from the ICG within the lymphatic vasculature was collected with an electron-multiplying charge-coupled device (EMCCD) (model 7827-0001, Princeton Instruments). Filter sets were used to reject backscattered and reflected excitation light. Images were acquired with V++ software (Total Turnkey Solutions, Sydney, Australia). The integration time for fluorescence images was 200 ms: 300–900 images were collected per lateral side per rat for lymph propulsive frequency measurements. Images were collected at or before day zero (when the first CIA injection was administered) and at days 11, 13, and 18 following CIA induction.

### Data analysis

NIRF images were loaded into ImageJ software (NIH), and fluorescence intensity values were quantified and imported into Microsoft Excel for computation of lymphatic pumping function as previously described [24]. Briefly, the number of forward lymphatic pulses minus those observed traveling in the distal direction in the hind limbs over a period of 5 minutes was computed and reported as a measure of lymphatic pumping function. The lymphatic vessels afferent to the popliteal LNs were interrogated. Quantification was done blinded. Statistical significance for comparison of the amount of etanercept delivered and LN uptake on the basis of μg/mL for the administration groups was determined from univariate and multivariate analysis of variance (ANOVA and MANOVA). Pairwise comparisons using contrast and the ANOVA model were used to compare the group differences for percent swelling and lymphatic pumping function, among the SOFUSA™, untreated, SC, and ID groups. These comparisons were performed separately for data collected at days 11, 13, and 18 post-CIA and the results can be used for examining influence of disease progression/regression upon measurements of swelling and lymphatic pumping function.

## Results

### PK and quantification studies show lymphatic delivery of etanercept via SOFUSA™

**Fig. 2:**
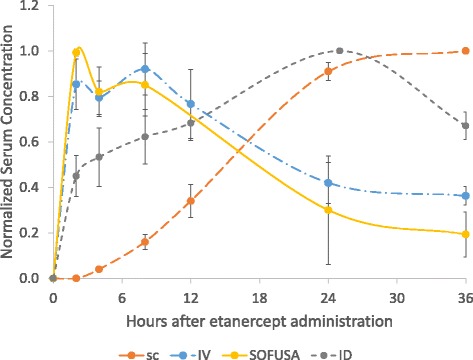
Normalized mean serum concentration of etanercept plus or minus standard deviation as a function of time following subcutaneous (*SC*), intradermal (*ID*), intravenous (*IV*), and SOFUSA^TM^ administration of 1 mg of drug

Figure 2 shows the early time course of normalized mean plus or minus the standard deviation of etanercept serum concentration normalized to peak concentration following 1 mg of etanercept delivery via SOFUSA™ and IV, SC, and ID administration. Table 1 shows the actual values at peak times, and the values at the 36-h time point. Consistent with past PK modeling studies of varying routes of antibody administration, the profile following IV administration shows initially high serum concentrations with moderately rapid clearance from the blood vasculature. In contrast, the PK profile associated with SC administration demonstrates a comparatively slower uptake into the blood vasculature with substantially lower serum values and reduced bioavailability [25] than found following the IV administration. In contrast to SC administration, the ID and SOFUSA™ PK profiles show rapid uptake from the intradermal space into the lymphatic plexus beneath the epidermis, and through the lymphatics, which empty into the blood vasculature. The SOFUSA™ PK profile shows more rapid uptake than the ID profile, due possibly to the larger area of administration (infusion area of 66 mm^2^ as compared to two ID injection sites of < 1 mm^2^) and enhanced update mechanisms that may be attributed to nanotopography. Due to the potential variability of the injection depth beneath the epidermis, ID administrations may penetrate too deeply, miss the initial lymphatics, and in part may mimic an SC injection, with loss of bioavailability.

**Table 1:** Mean plus or minus the SD of etanercept serum concentration (μg/mL) at the time of maximum concentration (in parentheses) and 36 h after administration

Route of administration	Mean ± SD maximum serum concentration of etanercept, μg/mL (time of maximum concentration)	Mean ± SD serum concentration of etanercept, μg/mL after 36 h administration
SOFUSA™ (*n* = 6)	18.28 ± 6.95 (2 h)	4.91 ± 3.76
IV (*n* = 6)	110.84 ± 15.03 (2 h)	47.06 ± 4.81
	119.62 ± 16.73 (8 h)	
ID (*n* = 6)	31.08 ± 5.68 (25 h)	20.65 ± 2.94
SC (*n* = 4)	13.50 ± 3.21 (24 h)	13.50 ± 3.21

*IV* intravenous, *ID* intradermal, *SC* subcutaneous

Despite the differences in serum PK profiles and values, Table 2 shows that the amount of etanercept delivered to the body was statistically identical with SOFUSA™, IV, SC, and ID administration, as determined through radioactive balance. These results suggest that the delayed and attenuated PK profiles from SC, ID, and SOFUSA™ administration at early time points arise from delayed input into the blood vascular compartment from the intradermal (from SOFUSA™ and ID administrations) and interstitial (from SC administration) tissue compartments.

**Table 2:** Amount of etanercept administered (mg) as determined from radioactivity balance

Route of administration	Dose ± SD of etanercept from radioactivity balance, mg
SOFUSA™ (*n* = 10)	1.07 ± 0.031
IV (*n* = 12)	0.96 ± 0.004
ID (*n* = 12)	1.00 ± 0.007
SC (*n* = 12)	0.91 ± 0.031

*IV* intravenous, *ID* intradermal, *SC* subcutaneous

#### SOFUSA^*TM*^ delivers material directly into the intradermal space drained by initial lymphatics

**Fig. 3:**
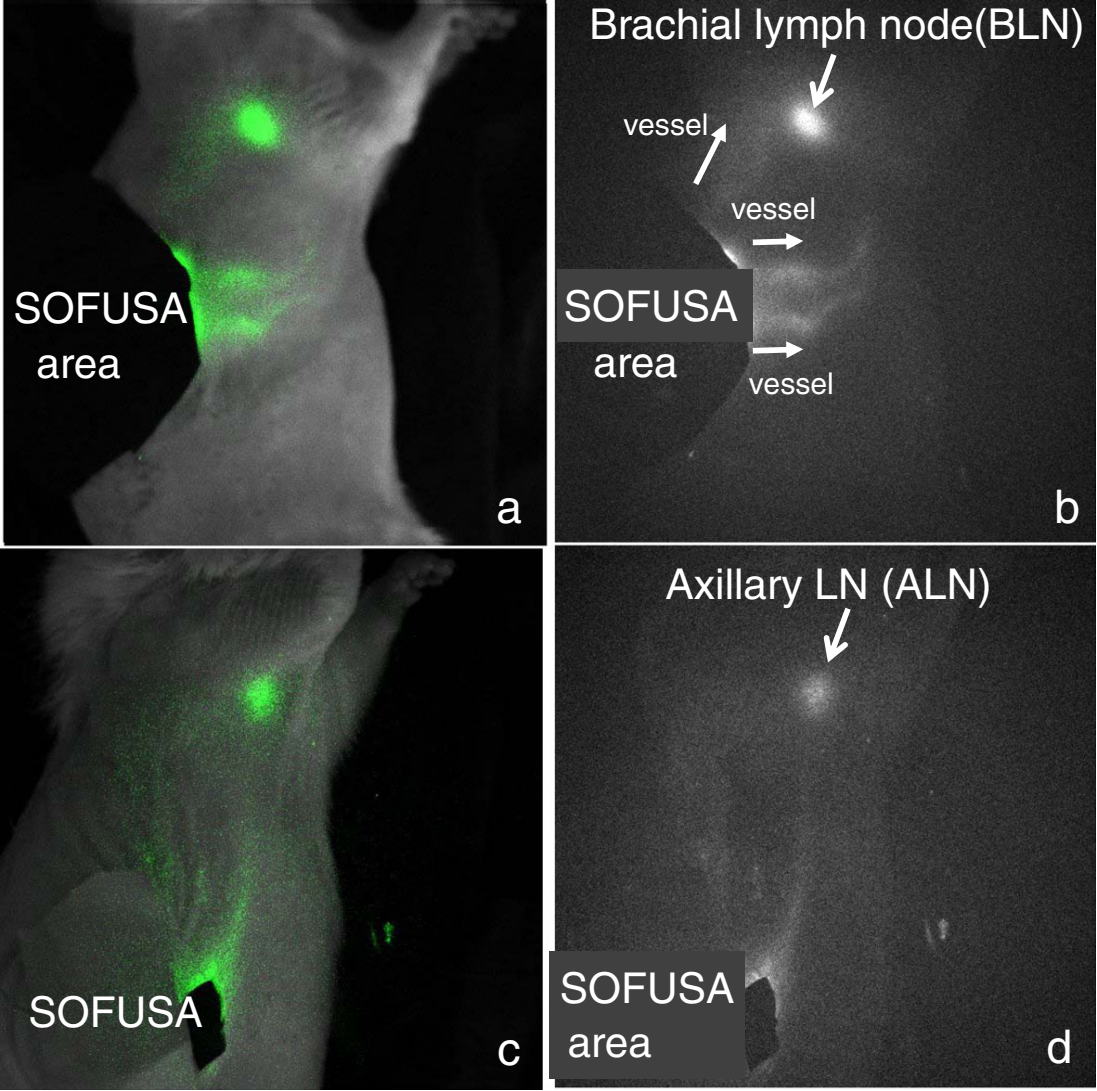
Near-infrared fluorescence lymphatic imaging of indocyanine green (ICG) delivered via SOFUSA^TM^ in normal rats showing different drainage patterns. **a** Fluorescence image shows drainage to brachial lymph nodes (BLNs) overlaid onto a white light image. **b** Fluorescence image alone (see Additional file 1: Video 1 showing lymphatic pumping of ICG delivered via SOFUSA^TM^ to the BLN). **c** Fluorescence image shows drainage to axillary lymph nodes overlaid onto a white light image. **d** Fluorescence image alone (see Additional file 2: Video 2 showing lymphatic pumping of ICG delivered via SOFUSA^TM^ to the BLN)

**Fig. 4:**
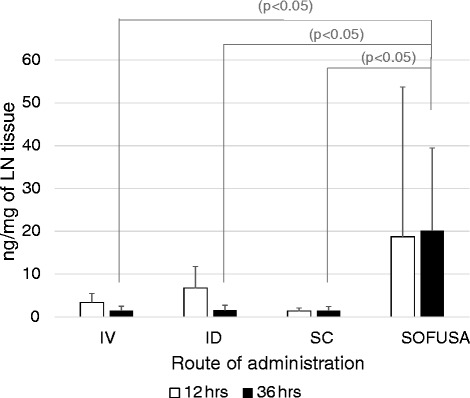
Mean ± SD ng/mg of etanercept in lymph nodes (*LNs*) with the greatest radioactivity in animals 12 and 36 h following etanercept administration via intravenous (*IV*), intradermal (*ID*), subcutaneous (*SC*), and SOFUSA^TM^ administration. Values of etanercept concentration following systemic IV administration represent the mean ± standard deviation of all LNs harvested. Values of etanercept concentration following ID, SC and SOFUSA^TM^ administration represent the mean ± standard deviation of the most radioactive LN harvested from each animal. Two-tailed *p* values denote significant differences between etanercept LN concentrations at 36 h following administration via the SOFUSA^TM^, IV, ID, and SC routes



Two approaches were utilized to track delivery via SOFUSA™. Figure 3 shows that the SOFUSA™ device delivers ICG directly into the intradermal space drained by the initial lymphatics for efficient uptake into the lymphatic vasculature, as shown by the highlighted lymphatic vessels, and the lymphatic pumping to draining LNs, shown in Additional file 1: Video 1 and Additional file 2: Video 2. Because SOFUSA™ administration provides local intradermal delivery that feeds into draining LN basins that vary widely between animals (see Additional file 3: Figure S1), we observed a large variation between radiolabeled etanercept delivery to right, left axillary/brachial and right, left inguinal LNs, often seeing one, but not all LNs, as radioactive. Likewise, variation in the levels of radiolabeled etanercept in regional draining LNs was also seen in SC and ID administration, but not in systemic IV administration, with which there were uniform but low tissue-concentrations of etanercept. To account for the different locoregional lymph drainage patterns between different animals, we compared etanercept concentration in the single LN with the highest radioactivity after administering radiolabeled etanercept via SOFUSA™ and the SC and ID route. Due to the more uniform systemic delivery to LNs following IV administration, we reported the mean radioactivity across all LNs at 12 and 36 h after administration. Figure 4 shows that SC and IV administration resulted in lower etanercept concentration in the LNs after 12 h as compared to SOFUSA™ and ID administration. In addition, the tissue concentration of etanercept in the LNs after 36 h was significantly greater (*p* < 0.05) when administered via SOFUSA™ than via the ID or SC routes, despite the potentially large variation in drainage routes, or via IV administration. The SOFUSA™ group variation was likely due to the larger area for infusion as compared to the SC and ID injection sites and to the drainage to one or more lymphatic basins.

**Additional file 1: Video 1.** Lymphatic pumping of ICG delivered via SOFUSA^TM^ to the brachial lymph node shown in Fig. 3. (AVI 9656.32 kb)




**Additional file 2: Video 2.** Lymphatic pumping of ICG delivered via SOFUSA^TM^ to the axillary lymph node shown in Fig. 3. (AVI 9861.12 kb)


This finding may be consistent with the PK results (Fig. 2, Table 1), showing that etanercept is predominantly located in the lymphatic vasculature following SOFUSA™ administration, with the smallest amount of drug in the systemic circulation at 36 h after administration when compared to IV, ID, and SC administration. Due to radioactivity half-life considerations, we were unable to determine the time at which clearance from draining LNs occurred.

Nonetheless, when taken together our results showing reduced blood serum concentration and increased concentration in draining LNs suggests there may be a mechanism by which etanercept administered by SOFUSA™ is retained within the lymphatic vasculature. Blood circulation times for therapeutic antibodies can be prolonged by enhanced neonatal Fc receptor (FcRN) binding, cellular uptake, and non-destructive recycling that occurs in epithelial and endothelial cells. FcRN expression has been identified in blood vascular endothelium [26] and elevated by pro-inflammatory cytokines TNF-α and IL-1β [27], but has not previously been identified in lymphatic endothelial cells. Using flow cytometry, we demonstrated FcRN expression in immortalized lymphatic endothelial cells (data not shown for brevity), further suggesting that FcRN-mediated retention may occur in the lymphatics as it does in the blood vasculature. It remains unknown whether FcRN expression in the lymphatics changes with inflammation and whether it may alter retention of etanercept.

### Etanercept administration into the lymphatics via SOFUSA™ delivery improves lymphatic pumping

Additional file 4: Video 3 and Additional file 5: Video 4 show examples of the sluggish flow and reflux in the lymphatic vessels afferent to the popliteal LNs in untreated rats that consistently occurred 18 days after CIA induction. Impaired lymphatic pumping function has also been visualized in the arms and legs of humans with peripheral vascular disease and lymphedema [28]. In contrast to Additional file 4: Video 3 and Additional file 5: Video 4, Additional file 6: Video 5 shows an example of lymphatic pumping of the lymphatic vessels afferent to popliteal LNs in animals treated by SOFUSA™ 18 days after CIA induction. It is noteworthy that we observed no differences between the groups in the lymphatic pump function in the large vessel efferent to the inguinal LN and afferent to the axillary LN. In addition, we found no significant correlation between percent swelling and the lymphatic pumping function of vessels afferent to the popliteal LNs measured at days 11, 13, and 18 in untreated animals, although these results do not rule out the expected relationship between lymphatic pump efficiency and onset of swelling.



**Additional file 4: Video 3.** Example of sluggish lymphatic pump function in the hind limb of an untreated rat 18 days post CIA induction. (AVI 2242.56 kb)




**Additional file 5: Video 4.** Example of forward and retrograde lymphatic pump function in the hind limb of an untreated rat 18 days post CIA induction. (AVI 1843.2 kb)




**Additional file 6: Video 5.** Example of lymphatic pumping function in the hind limb of a SOFUSA^TM^-treated rat 18 days post CIA induction. (AVI 2027.52 kb)


**Fig. 5:**
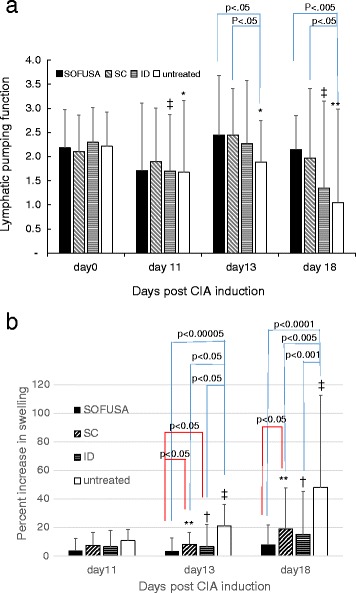
**a** Mean ± SD of lymphatic pumping function as a function of time after induction of collagen-induced arthritis (*CIA*) in the four groups: untreated (*white bars*), treated by SOFUSA™ (*solid bars*) and treated by the subcutaneous (*SC*) (*diagonal lined bars*), and intradermal (*ID*) (*horizontal lined bars*) routes. Two-tailed, pairwise test *p* values in the framework of the analysis of variance (ANOVA) model at days 13 and 18 after CIA induction (*bars* connecting the different groups) show significant reduction in lymphatic pumping function in the untreated groups compared to SOFUSA™ and SC administration groups: significant decrease (*p < 0.005, **p <0.05, paired Student’s *t* test, one-tailed) in lymphatic pumping in untreated animals at 11, 13, and 18 days after CIA induction compared to baseline (day 0); significant decrease (^‡^ *p* < 0.005, paired Student’s *t* test, one-tailed) in lymphatic pumping at 11 and 18 days after CIA induction in animals treated with ID administration of etanercept as compared to baseline (day 0). **b** Mean ± SD of percent increase in swelling as a function of time after CIA induction in the four groups: untreated (*white bars*), and treated by SOFUSA (*solid bars*) and by the SC (*diagonal lined bars*), and ID (*horizontal lined bars*) routes. Pairwise *F* test *p* values using contrast in the ANOVA model at days 13 and 18 after CIA induction (*bars* connecting the different groups) denote significant reduction in swelling in the SOFUSA™ and SC administration groups when compared to the untreated group: **p < 0.005 for significant increase in swelling at day 18 compared to day 13 in animals treated with SC administration; ^†^ *p* < 0.05 for significant increase in swelling at day 18 compared to day 13 in animals treated with ID administration; ^‡^ *p* < 0.005 for significant increase in swelling at day 18 compared to day 13 in untreated animals

Figures 5a and b shows the histogram of the lymphatic pumping function and percent swelling in both hind limbs of each animal 11, 13, and 18 days after CIA induction in the untreated groups, and in those treated with etanercept administered by the ID and SC route and by SOFUSA™. No animals were excluded from the analysis, even those that had minimal swelling. At days 13 and 18 post CIA induction, univariate analyses showed: (1) statistically significant greater lymphatic pumping in the hind limbs of animals treated with SOFUSA™ and SC administrations than in untreated animals and (2) significant reduction in swelling of the hind limbs of all treated groups when compared to untreated animals. In addition, animals treated by SOFUSA™ had significantly reduced hind limb swelling at days 13 and 18 when compared to SC-treated animals.

Paired group comparisons using contrast in the ANOVA model showed that the lymphatic pumping function was significantly decreased with progression of disease in untreated animals and swelling was significantly increased with progression of disease in untreated animals and animals treated by SC and ID administration. No statistically significant increase in swelling was observed in animals treated by SOFUSA™ administration.

Simultaneous comparison of both metrics of swelling and lymphatic pumping function were performed using Wilk’s lambda test, with the level of significance determined (*p* = 0.008 after Bonferroni adjustment). Multivariate analyses (Table 3) showed that there were no significant differences between the SOFUSA™ and all other treated groups based upon the combination of percent swelling and pumping function comparisons at day 11. However on day 13 and 18, all treated groups were statistically different compared to untreated animals.

**Table 3:** Multivariate *p* values to assess the differences in swelling and lymphatic pumping function at days 11, 13, and 18 in animals with etanercept administered by SOFUSA™ and the SC and ID routes and in untreated animals

	ID	SC	Untreated
Day 11 (overall, *p* = 0.62)			
SOFUSA™	0.17	0.43	0.84
ID		0.70	0.35
SC			0.76
Day 13 (overall, *p* = 0.0002)			
SOFUSA™	0.07	0.39	<0.0001
ID		0.69	0.0017
SC			0.0015
Day 18 (overall, *p* <0.0001)			
SOFUSA™	0.48	0.10	<0.0001
ID		0.13	0.0006
SC			0.004

ID intradermal, SC subcutaneous. *P* < 0.008 is statistically significant due to application of the Bonferroni adjustment for multiple comparisons

## Discussion

In this work we showed that the function of afferent lymphatic vessels in the hind limbs of rats with CIA-induced arthritis declined with disease progression, but may be reversed through SOFUSA™ administration of etanercept to draining inguinal and axillary LNs, consistent with the hypothesis that the lymphatic system mediates inflammatory-erosive arthritis [5, 6]. Furthermore, we showed reduced swelling with etanercept administration to the lymphatics, via the nanotopography device (SOFUSA™) compared to administration via the clinically used SC route.

It is noteworthy that prior studies have employed IV and SC administration of etanercept to effect changes in swelling in CIA animals [29]. Systemic intraperitoneal (IP) administration of anti-CD20 (depletion of B cells) has also been used to effect decreased knee synovium volume, enhanced afferent lymphatic pumping function, and macrophage egress from inflamed joints in TNF-Tg mice, presumably by “unclogging” the draining LN through removal of B cells [11]. Because our work shows improvement in afferent lymphatic function and reduced swelling with SOFUSA™ delivery over that of SC administration of etanercept, one could speculate that direct lymphatic administration of anti-CD20 therapy to reach B cells in draining LNs could result in improved response with lower dose, reduced systemic exposure, and lower incidence of adverse events. Another mechanism that may be responsible for impairment of afferent lymph drainage could be the well-known neutrophil recruitment to subcapsules of draining LNs in inflamed peripheral tissues [30]. Recently it has been shown that RA patients have elevated numbers of neutrophils that participate in the process of NETosis induced by pro-inflammatory cytokines, including TNF-α. NETosis occurs when neutrophils spew DNA nets that display autoantigens [31] and can intravascularly trap cells and cellular debris to play a critical role in hemovascular thrombi formation [32].

Whether NETosis can impair afferent lymph draining in the synovial fluid of RA patients and whether TNF-α antagonists delivered into the lymphatics can restrict NETosis or other immune processes that impact drainage, remains to be answered. Other biologics that target the immune system could be delivered into the lymphatics for regional treatment of localized autoimmune skin diseases through direct targeting of the immune system. In addition, stimulation of the immune system using systemic IV administration of biologics to block checkpoint inhibition may be less effective when compared to SOFUSA™ or ID-directed lymphatic delivery, which can directly access LNs at the sites where immune cell activation is induced [33]. The presence of FcRN receptor expression on the lymphatic endothelium may prolong the presence of therapeutic biologics in the lymphatic vasculature that feeds draining LNs. It is also noteworthy that SOFUSA™ resulted in unilateral delivery to draining inguinal and axillary LNs, not direct delivery to the bilateral popliteal LNs draining the inflamed hind limbs. Future work is needed to understand the interaction of biologics with the lymphatic endothelium and LN stroma and whether the “abscopal” effect, i.e., the well-known distant immune responses observed away from the site of cancer treatment [34], may play a role in RA treatments. The hypothesis that delivery of anti-TNF to inguinal or axillary LNs can efficiently suppress the progression of systemic disease, thus reducing joint swelling and preserving lymphatic pumping in other anatomical locations, needs to be further explored.

However, it also important to note that while ID or SOFUSA™ administration affords delivery through draining LNs, the lymphatics *do* empty into the blood vasculature, enabling systemic delivery, as evidenced by the early PK data presented herein. While SC, IP, and IV routes of administration provide systemic delivery into the blood circulation by which biologics could enter the lymphatics via high endothelial venules in LNs, administration into the intradermal space via ID or SOFUSA™ delivery may enable systemic delivery through regional lymphatics with maximal exposure to draining LNs.

In the clinic, ID injection may be irrelevant because: (1) the intradermal space is too small to receive therapeutic drug volumes and (2) advanced skill is required for successful ID injection. However, it is relevant to compare ID injection with SOFUSA™ infusion, because the device may mimic drug placement in the intradermal space of a successful ID injection and could potentially infuse a therapeutic dose into the intradermal space in humans. Indeed, it may be surprising in this animal study that ID administration did not impact the lymphatic pumping function and swelling as well as the nanotopography device did. This observation could arise from accidental penetration of conventional needles beyond the initial lymphatics, where lymphatic uptake is most efficient. Indeed, in our NIRF imaging studies conducted in humans (for review see [18]), we often observed operator error in performing the intradermal injections: if the Mantoux procedure is not accurate and a weal is not formed on the skin, there is considerable delay (10–60 minutes) until the partial lymphatic uptake of ICG enabling lymphatic imaging. Another reason for the improved performance of nanotopography infusion over ID administration may be the enhanced area of SOFUSA™ microneedle coverage and the enhanced disruption of tight junctions through mechanotransduction mechanisms [20] that could facilitate lymphatic uptake. Compared to SOFUSA™ administration, the delayed PK profile and reduced radiolabeled etanercept uptake into draining LNs (Figs. 2 and 4) after ID administration are consistent with improved lymphatic uptake with nanotopographic delivery.

There have been several academic and commercial efforts to develop microneedle-based delivery systems [35–37], but this is the first to demonstrate improved pharmacodynamics over conventional Mantoux procedures. It is noteworthy that, unlike other studies that demonstrate efficacy in animals with at least a 50% increase in swelling in one hind limb [29], we conservatively included all animals in our study, including those with minimal swelling. While there are several rodent models of RA, the CIA model is a commonly used to mimic both the innate and adaptive immune responses that are important in RA progression. When used in combination with adjuvant-induced arthritis, and genetic models (such as TNF-Tg mice), the CIA rat model provides an efficient means for predicting therapeutic efficacy in humans [38]. Since completing our work, another study in TNF-Tg animals has shown that systemic IP administration of anti-TNF drugs increase the lymphatic pump [14], in agreement with our data on early CIA progression in rats treated with etanercept versus untreated CIA rats. Extension of this work to adjuvant-induced arthritis, genetic models of RA, and human RA patients remains to be shown.

Finally, there are limitations to the study presented herein. First, we are unaware of a commercially available isotype control protein that is identical to etanercept but with abrogated TNF binding. It is possible that the Fc portion of etanercept could itself evoke an immune response within the lymphatics, necessitating further study of the interaction with biologics within the lymphatic vasculature. Another limitation for quantifying lymphatic pumping function using our approach should be noted. Because we quantified the pumping function as the number of proximally propelled minus the number of distally propelled lymph “packets” imaged by NIRF, our metric may not discriminate between: (1) the arrest of lymphatic pumping and (2) a strong lymphatic pump that pumped and refluxed lymph equally in both proximal and distal directions. A more suitable metric of lymphatic efficiency from NIRF imaging needs to be developed that might better predict swelling and response to therapy. Nonetheless, these preclinical studies demonstrate modification of the lymphatic pump function in a CIA animal model and suggest that future work to therapeutically mediate lymphatic function through nanotopography delivery of immune targets could result in more effective treatments for RA.

## Conclusion

Administration of etanercept into the draining lymphatics of inflamed joints significantly reduced swelling and improved lymphatic pumping when administered using nanotopography (SOFUSA™). This work suggests that alternative routes of locoregional administration could provide more effective delivery than SC or IV administration.

## Additional files

Additional file 3: Figure S1. Variation of lymphatic pathways draining to inguinal lymph nodes (*ILN*) following ICG injection (*yellow dotted arrow*) with conventional needle on the dorsal surface of the rat. (PDF 146 kb)

## Abbreviations

ANOVA: Analysis of variance
CIA: Collagen induced arthritis
ELISA: Enzyme-linked immunosorbent assay
FcRN: Neonatal Fc receptor
ICG: indocyanine green
ID: Intradermal
IL-1: Interleukin-1
IL-6: Interleukin-6
iNOS: Inducible nitric oxide synthase
IP: Intraperitoneal
IV: Intravenous
LN: Lymph node
NIRF: Near-infrared fluorescence
PBS: Phosphate-buffered saline
PK: Pharmacokinetic
RA: Rheumatoid arthritis
SC: Subcutaneous
TNF: Tumor necrosis factor
TNF-Tg: Tumor necrosis factor transgenic
