# Supplementary figures and images for: Lymphatic delivery of etanercept via nanotopography improves response to collagen-induced arthritis
Source: Arthritis Res Ther. 2017 May 31;19:116. doi: 10.1186/s13075-017-1323-z (PMC5452411; doi:10.1186/s13075-017-1323-z)

Figure S1

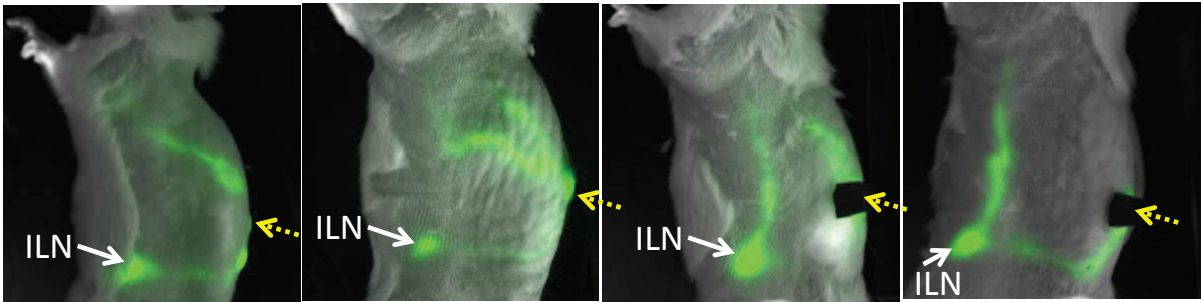

Supplement: Supplementary file 6 — Variation of lymphatic pathways draining to inguinal lymph nodes (ILN) following ICG injection (yellow dotted arrow) with conventional needle on the dorsal surface of the rat. (PDF 146 kb) [file 13075_2017_1323_MOESM3_ESM.pdf]
